# Advancing mid‐rectal cancer surgery: Unveiling the potential of natural orifice specimen extraction surgery in comparison to conventional laparoscopic‐assisted resection

**DOI:** 10.1002/cnr2.2003

**Published:** 2024-05-04

**Authors:** Shan Muhammad, Zheng Jiang, Tao Fan, QingChao Tang, Yang Hai, Sundas Bint E. Ehsan, Maimoona Bilal, Albina A. Zubayraeva, YiBo Gao, Jie He

**Affiliations:** ^1^ Department of Thoracic Surgery National Cancer Center/National Clinical Research Center for Cancer/Cancer Hospital, Chinese Academy of Medical Sciences and Peking Union Medical College Beijing China; ^2^ Laboratory of Translational Medicine, National Cancer Center/National Clinical Research Center for Cancer/Cancer Hospital Chinese Academy of Medical Sciences and Peking Union Medical College Beijing China; ^3^ Department of Colorectal Surgery National Cancer Center/National Clinical Research Center for Cancer/Cancer Hospital, Chinese Academy of Medical Sciences & Peking Union Medical College Beijing China; ^4^ Department of Colorectal Surgery The Second Affiliated Hospital of Harbin Medical University Harbin China; ^5^ Department of Children's and Adolescent Health Public Health College of Harbin Medical University Harbin China; ^6^ Department of General Surgery Second Affiliated Hospital of Chongqing Medical University Chongqing China; ^7^ Department of General Surgery I.M. Sechenov Affiliated Hospital of I.M. Sechenov First Moscow State Medical University (Sechenov University) Moscow Russia

**Keywords:** conventional laparoscopic‐assisted resection, long‐term outcomes, mid‐rectal cancer, natural orifice specimen extraction surgery, postoperative recovery, quality of life

## Abstract

**Background:**

Mid‐rectal cancer treatment traditionally involves conventional laparoscopic‐assisted resection (CLAR). This study aimed to assess the clinical and therapeutic advantages of Natural Orifice Specimen Extraction Surgery (NOSES) over CLAR.

**Aims:**

To compare the clinical outcomes, intraoperative metrics, postoperative recovery, complications, and long‐term prognosis between NOSES and CLAR groups.

**Materials & Methods:**

A total of 136 patients were analyzed, with 92 undergoing CLAR and 44 undergoing NOSES. Clinical outcomes were evaluated, and propensity score matching (PSM) was employed to control potential biases.

**Results:**

The NOSES group exhibited significant improvements in postoperative recovery, including lower pain scores on days 1, 3, and 5 (p < .001), reduced need for additional analgesics (p = .02), shorter hospital stays (10.8 ± 2.3 vs. 14.2 ± 5.3 days; p < .001), and decreased intraoperative blood loss (48.1 ± 52.7 mL vs. 71.0 ± 55.0 mL; p = .03). Patients undergoing NOSES also reported enhanced satisfaction with postoperative abdominal appearance and better quality of life. Additionally, the NOSES approach resulted in fewer postoperative complications.

**Conclusion:**

While long‐term outcomes (overall survival, disease‐free survival, and local recurrence rates) were comparable between the two methods, NOSES demonstrated superior postoperative outcomes compared to CLAR in mid‐rectal cancer treatment, while maintaining similar long‐term oncological safety. These findings suggest that NOSES could serve as an effective alternative to CLAR without compromising long‐term results.

## 1 INTRODUCTION

Rectal cancer continues to be a significant health burden, with about 40 000 new cases diagnosed in the United States each year, accounting for approximately one‐third of all colorectal cancers.^1^ The therapeutic approach to mid‐rectal cancers has historically posed unique challenges due to its anatomical position and proximity to vital structures.^2^ In this context, two minimally invasive surgical techniques, natural orifice specimen extraction surgery (NOSES) and conventional laparoscopic‐assisted resection (CLAR), have gained considerable attention. Both offer the potential for reduced morbidity, faster recovery, and improved quality of life compared to traditional open resection methods.^3^, ^4^

NOSES is a form of transanal minimally invasive surgery, aiming to reduce abdominal incisions by extracting the surgical specimen through a natural orifice, such as the anus, vagina, or mouth.^4^ This approach can potentially result in decreased postoperative pain, a reduced risk of wound complications, and improved cosmesis.^5^ Conversely, CLAR has long been a mainstay of minimally invasive surgery for mid‐rectal cancer. It has demonstrated superior outcomes compared to open surgery in terms of lower blood loss, reduced postoperative pain, and shorter hospital stays.^6^, ^7^, ^8^ Notwithstanding, the operation still necessitates an abdominal incision for specimen extraction, potentially leading to wound‐related complications.^9^ With an ever‐growing emphasis on improving surgical outcomes and patient satisfaction, there has been a progressive shift toward minimally invasive procedures in colorectal surgery.^10^ The advent of NOSES is a testament to this evolving landscape, potentially reducing surgical trauma and further improving patient quality of life by obviating the need for abdominal incisions, traditionally needed for specimen retrieval.^11^

While NOSES presents promising benefits, its adoption in managing mid‐rectal cancer remains restrained, largely influenced by apprehensions over oncological security, procedural intricacies, and the proficiency of the surgeon.^12^, ^13^ Consequently, CLAR, recognized for its consistent safety, effectiveness, and methodological standardization, remains the predominant choice in addressing mid‐rectal cancers.^14^ In our prior research, we meticulously compared NOSES and CLAR for the treatment of low rectal cancer.^15^ The present investigation shifts the focus to a comparison of these surgical techniques in the context of mid‐rectal cancers.

In this context, the current study seeks to bridge this knowledge gap by comprehensively comparing the treatment effects of NOSES and CLAR in mid‐rectal cancer management. Through this comparative study, we aim to shed light on these surgical approaches' relative merits, potentially informing surgical decisions and shaping future research in the field.

## 2 MATERIALS AND METHODS

### 2.1 Selection of enrolled patients

Between January 2013 and December 2017, all the patients diagnosed with mid‐rectal cancer and undergone radical resection at the Department of Colorectal Surgery, National Cancer Center/National Clinical Research Center for Cancer/Cancer Hospital, Chinese Academy of Medical Sciences & Peking Union Medical College, Beijing, China, were included in this study. Patients were then divided into two groups based on the surgical procedure they underwent: the NOSES group or the CLAR group. All surgeries were performed by a highly skilled, unified team, each member of which had previously carried out a minimum of 100 cases of laparoscopic rectal cancer resections.

In our rigorous preoperative evaluations, including imaging studies like CT scans and MRI, we meticulously checked for and excluded patients with distant metastasis. This selection process was pivotal in maintaining the integrity of our comparative analysis between NOSES and CLAR groups.

### 2.2 Inclusion criteria

The criteria for inclusion in our study were carefully delineated to ensure robust data collection. We included patients aged between 18 and 80 years who had a histopathological confirmation of mid‐rectal cancer. This was identified when the distance from the tumor's lower edge to the dentate line fell within the range of 5 cm to 10 cm. Further, we confirmed that the diameter of the tumor was ≤3 cm and the T stage was within T3 through preoperative imaging examinations, including CT and MRI scans. Moreover, we included patients with a body mass index (BMI) of ≤30 kg/m^^2^.

### 2.3 Exclusion criteria

We also specified explicit exclusion criteria to maintain the study's integrity. Patients were not eligible for inclusion if they presented contraindications for laparoscopic surgery or required emergency surgery due to acute intestinal obstruction, perforation, or bleeding. Additionally, we excluded patients diagnosed with other primary malignant cancers in different organs, those who had undergone prophylactic ostomy or ostomy for other reasons, and patients with multiple primary colorectal cancer. We also excluded patients who had accepted preoperative radiotherapy or chemotherapy, those with a previous medical history of cancer, and those instances where incomplete information or lost follow‐up data was recorded.

Based on the aforementioned selection criteria, a total of 136 patients were ultimately included in this study, with the NOSES group comprising 44 patients and the CLAR group consisting of 92 patients.

### 2.4 Ethical consideration

Before proceeding with surgery, a multidisciplinary team (MDT) composed of an endoscopist, an anesthetist, and two surgeons extensively explained both the NOSES and CLAR procedures to the patients. Patients then had the autonomy to decide their treatment course based on these consultations. We ensured to obtain informed consent from all patients regarding the use of their treatment‐related data for research purposes. Additionally, we guaranteed that the collection of this data would not influence the quality of treatment they received, and their private information would be kept strictly confidential. The Ethics Committee of the National Cancer Center/National Clinical Research Center for Cancer/Cancer Hospital, affiliated with the Chinese Academy of Medical Sciences & Peking Union Medical College in Beijing, China (CAMS), granted approval for this study. The ethical approval number for this study is (Ethics Committee 17–116/1439). In this study, due to its analytical nature, a separate ethics review was not required. All procedures and methodologies were in strict adherence to the ethical standards of our institution and conformed to the Declaration of Helsinki, as revised in 2013.

### 2.5 Surgical technique

**FIGURE 1:**
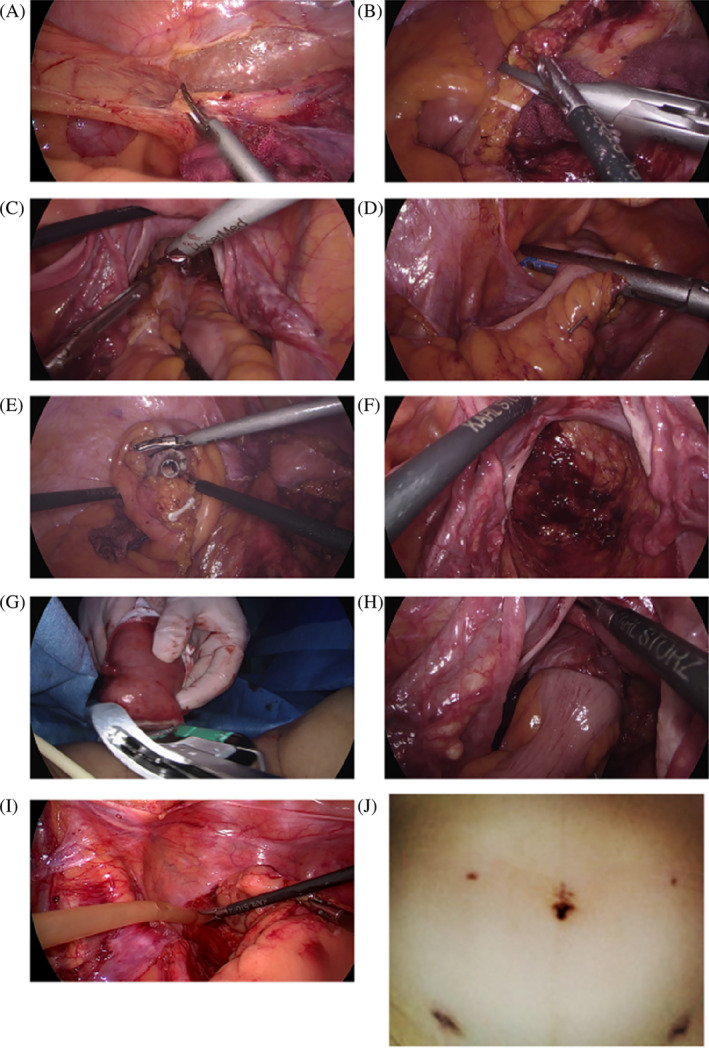
Intra‐operative illustration of the NOSES surgical procedure. (A) Mobilization of the Descending and Sigmoid Colon. (B) Clipping of the inferior mesenteric artery using hem‐o‐lock clips. (C) Dissection of the mesorectum following total mesorectal excision (TME). (D) Delivery of the anvil in the sigmoid and resection of the rectum using the linear stapler echelon 60. (E) Removal of the anvil connection rod at the distal end of the sigmoid colon. (F) transanal extraction of the opened distal rectal stump for smooth specimen extraction. (G) Extracorporeal resection of the rectal specimen and inspection for adequate distal margin. (H) Passage of the head of the circular stapler through the anal orifice, followed by an end‐to‐end double‐stapled anastomosis. (I) positioning of the post‐operative peritoneal drainage tube near the anastomosis site. This figure provides a detailed intra‐operative depiction of the NOSES surgical procedure, illustrating the key steps involved in this minimally invasive approach to mid‐rectal surgery. (J) Three‐week postoperative assessment revealed favorable cosmetic results on the abdominal wall. Notably, the absence of additional incisional scars for specimen extraction was evident. Instead, five inconspicuous trocar scars were observed, contributing to excellent postoperative esthetic outcomes.

The surgical approach utilized for the NOSES group has been comprehensively elaborated in our prior work.^11^ Yet, to cater to the context of this investigation, a concise overview is presented below. Additionally, Figure 1 offers an illustrative depiction of the NOSES surgical procedure. For a more detailed and dynamic visual understanding, supplementary videos of each major step in the NOSES surgical procedure are available in the Supplementary Materials section (Supplementary Material 1).

Patients underwent bowel preparation with oral laxatives and enemas. A single dose of either second‐generation Cephalosporins or Levofloxacin was administered intravenously during anesthetic induction, with repetition if surgery surpassed 3 hours. Post‐anesthesia, patients were positioned supine in a modified lithotomy stance. A five‐trocar laparoscopic method was employed. The tumor's location was then confirmed by a combination of digital rectal examination and intraoperative colonoscopy. The medial to lateral approach was utilized for mobilizing the descending and sigmoid colon, ensuring the preservation of the left gonadal vessels, left ureter, and inferior mesenteric nerve plexus. The inferior mesenteric artery was clipped using Hem‐o‐Lock clips (Teleflex Inc., USA) and transected. Lymph node dissection occurred at the root of the inferior mesenteric vessels. The mesorectum was dissected following the total mesorectal excision (TME) technique and was irrigated with 1% povidone‐iodine solution (Betadine®). The rectum was transected using a linear stapler (Echelon 60) 2‐3 cm below the tumor's lower edge. The distal rectum was then irrigated and incised for transanal specimen extraction. A sterile protective sleeve facilitated the specimen's extraction, ensuring no tumor deposition. The specimen was inspected extracorporeally, ensuring an adequate distal margin. The anvil of the circular stapler (CDH29) was introduced, followed by a purse‐string suture with 2–0 Prolene. The rectal stump was sealed with an Endo‐GIA linear stapler (AST45) and the specimen was retrieved. An end‐to‐end double stapled anastomosis was executed intracorporeally under laparoscopic guidance. Post‐operation, a peritoneal drainage tube was positioned near the anastomosis. The specimen's extraction through a natural orifice precluded the need for any additional abdominal incisions, thereby minimizing the risk of wound‐related complications.

Postoperatively, the exhaust time, which is the time to first passage of flatus or first bowel movement following surgery, was recorded. We also measured postoperative pain using the Visual Analogue Scale, which graded the pain intensity from 0 (no pain) to 10 (maximal pain ever experienced).^16^

Conversely, the CLAR group underwent conventional laparoscopic‐assisted resection, wherein an abdominal incision was necessary for the specimen's extraction. This was followed by the same postoperative protocols as in the NOSES group.

By comparing these techniques, our study aims to highlight the advantages and disadvantages of each, thereby enabling a more informed choice of treatment for patients with mid‐rectal cancer.

### 2.6 Data collection and follow‐up

To create a robust comparative analysis of NOSES and CLAR in the treatment of mid‐rectal cancer, a comprehensive data collection process was conducted, capturing a breadth of preoperative, intraoperative, postoperative, and pathological variables.

For this study, data collection and follow‐up procedures were conducted meticulously to ensure a comprehensive evaluation and accurate analysis of outcomes in patients with mid‐rectal cancer. The medical record system of CAMS, served as the primary source of information. The data collection process involved capturing both basic patient information and treatment‐related details. Basic patient information, including demographics and clinical characteristics, was obtained from medical records. Additionally, treatment‐related information such as surgical approaches (CLAR or NOSES) and the presence of incision‐related complications was recorded. Incision‐related complications encompassed postoperative bleeding, fever, infection, fat liquefaction within 1 day to 2 weeks postoperatively, and incisional hernia development within 3 months postoperatively. To assess patients' perceptions of their physical appearance and satisfaction with the scar, a Body Image Questionnaire (BIQ) was administered at 1 month after surgery.^17^ The BIQ, as outlined in Supplementary Material 2, provided insights into patients' attitudes toward their physical appearance. The impact of surgery on lower urinary tract, lower gastrointestinal tract, and pelvic organ prolapse symptoms was evaluated using the Pelvic Floor Distress Inventory‐20 (PFDI‐20) before surgery and at 3 months postoperatively.^18^ This assessment aimed to measure the extent to which these symptoms affected patients' quality of life (QoL).^18^ Additionally, anal function impairment in postoperative patients was assessed at 6 months postoperatively using the Wexner Incontinence Score, as outlined in Supplementary Material 3.^19^ The European Organization for Research and Treatment (EORTC) Quality of Life Measurement Scale System for Cancer Patients includes the EORTC QLQ‐C30,^20^ which was employed to evaluate patients' QoL at 3 months after surgery. This scale provides insights into various aspects of patients' QoL, serving as a valuable measure in assessing treatment outcomes.^20^

Follow‐up visits were scheduled at regular intervals to monitor patient progress and gather long‐term data. Patients who underwent surgery in our department were followed up every 3 months for the first 2 years after surgery, followed by 6‐month intervals for the subsequent 3 years or until the end of the study. In cases where patients did not return for in‐person follow‐up, information was obtained through communication via letter or telephone. The follow‐up period extended until patient death or the conclusion of the study in December 2022, ensuring comprehensive monitoring of patients throughout the study duration.

In line with the NOSES treatment philosophy, our study did not employ protective ileostomies in any of the patients, adhering to the approach of minimizing additional abdominal incisions. This decision is supported by current evidence suggesting that while ileostomies may reduce the clinical impact of anastomotic leakage, they do not prevent it and introduce additional complexities, such as the need for subsequent closure surgeries and extended hospital stays.^21^, ^22^, ^23^

By conducting thorough data collection and implementing a robust follow‐up process, this study aims to provide comprehensive insights into the outcomes and long‐term effects of mid‐rectal cancer treatment in the CLAR and NOSES groups.

### 2.7 Statistical analysis

Our statistical analysis involved propensity score matching (PSM) to balance the baseline characteristics between the NOSES and CLAR groups. Scores were computed from gender, age, BMI, ASA score, preoperative CEA, T stage, N stage, and preoperative PFDI‐20 scores. Logistic regression was used for variable assignment in the baseline data, and a caliper value of 0.2 was maintained. Quantitative data, expressed as mean ± SD, were analyzed using Student's t‐test and two‐factor repeated measures ANOVA for continuous data, including postoperative VAS scores. Categorical variables were analyzed with the Chi‐square test or Fisher's exact test, as appropriate. Further, survival analysis was conducted using the Kaplan–Meier method, with the log‐rank test to compare survival differences, and a Cox proportional hazards model to identify independent prognostic factors. The statistical significance level was set at *p* < .05. All analyses were carried out using R software (version 4.3.1) and GraphPad Prism (version 10.0.1).

## 3 RESULTS

### 3.1 Preoperative baseline and propensity‐score matching

A total of 136 patients with mid‐rectal cancer were included in our analysis, with 92 patients in the CLAR group and 44 patients in the NOSES group. In this investigation, we meticulously assessed and contrasted the preoperative baseline characteristics between the two groups. Analysis revealed no significant discrepancies in terms of gender, age, body mass index (BMI), American Society of Anesthesiologists (ASA) score, preoperative carcinoembryonic antigen (CEA), T stage, N stage, and preoperative pelvic floor distress inventory‐short form 20 (PFDI‐20) scores between the CLAR and NOSES groups (*p* > .05). Following propensity score matching (PSM), a subset of 44 patients was selected from each group to ensure a balanced representation of baseline attributes (*p* > .05), as detailed in Table 1.

**TABLE 1:** Baseline characteristics of mid‐rectal cancer patients in NOSES and CLAR groups.

	Before PSM			After PSM		
Characteristics	CLAR (*N* = 92)	NOSES (*N* = 44)	*p* value	CLAR (*N* = 44)	NOSES (*N* = 44)	*p* value
Gender (*N*, %)			.23			.53
Male	54 (58.7%)	21 (47.7%)		20 (45.5%)	21 (47.7%)	
Female	38 (41.3%)	23 (52.3%)		24 (54.5%)	23 (52.3%)	
Age (years)	58.3 ± 10.5	58.9 ± 11.6	.85	58.9 ± 10.2	58.9 ± 11.6	.75
BMI (kg/m^2^)	22.7 ± 3.0	22.8 ± 2.9	.54	22.8 ± 3.1	22.8 ± 2.9	.79
ASA grade (*N*, %)			.62			.79
I/II	75 (81.5%)	36 (81.8%)		35 (79.5%)	36 (81.8%)	
III	17 (18.5%)	8 (18.2%)		9 (20.5%)	8 (18.2%)	
Preoperative CEA (*N*, %)			.74			.76
Positive	17 (18.5%)	8 (18.2%)		8 (18.2%)	8 (18.2%)	
Negative	75 (81.5%)	36 (81.8%)		36 (81.8%)	36 (81.8%)	
T Stage (*N*, %)			.37			.64
Tis/T1	17 (18.5%)	11 (25.0%)		8 (18.2%)	11 (25.0%)	
T2	25 (27.2%)	16 (36.4%)		15 (34.1%)	16 (36.4%)	
T3	50 (54.3%)	17 (38.6%)		21 (47.7%)	17 (38.6%)	
N Stage (*N*, %)			.68			.67
N0	66 (71.7%)	32 (72.7%)		31 (70.5%)	32 (72.7%)	
N1/N2	26 (28.3%)	12 (27.3%)		13 (29.5%)	12 (27.3%)	
Preoperative PFDI‐20	7.08 ± 2.03	7.04 ± 1.71	.71	7.00 ± 1.97	7.04 ± 1.71	.82

*Note*: Before PSM: Patient data prior to propensity score matching. After PSM: Patient data following propensity score matching. BMI: Body Mass Index, a measure of healthy weight calculated from height and weight. ASA Score: American Society of Anesthesiologists score, assessing patient fitness before surgery. Values listed under ‘Age,’ ‘BMI,’ ‘ASA Score,’ and ‘Distance from anal verge’ represent averages with standard deviations (SD) indicating group variations. Percentages are calculated based on the total number of patients in each group before and after propensity score matching. Tumor size represents the average tumor size in patients with SD. Statistical Significance: The *p*‐value was calculated using appropriate statistical tests (independent‐samples T test, Mann–Whitney U test, Chi‐square test, or Fisher's exact test). *p*‐values <.05 are considered statistically significant.

### 3.2 Operative details: A comparative look

In terms of surgical factors, there were no statistically significant differences in operative time between the CLAR and NOSES groups (*p* = .68). However, a significant difference was observed in blood loss (*p* = .03), with the CLAR group experiencing an average blood loss of 71.0 ± 55.0 mL compared to 48.1 ± 52.7 mL in the NOSES group. The length of abdominal incision also exhibited a significant difference between the two groups (*p* < .001), with the CLAR group having an average incision length of 5.7 ± 0.7 cm, while the NOSES group had a significantly shorter incision length of 1.2 ± 0.2 cm. There were no statistically significant differences in the number of dissected lymph nodes (*p* = .32), the number of positive lymph nodes (*p* = .56), and positive margins between the two groups. No intraoperative complications were reported in either group (Table 2).

**TABLE 2:** Comparative analysis of postoperative outcomes for NOSES and CLAR in mid‐rectal cancer patients.

Outcome	Before PSM CLAR (*N* = 92)	NOSES (*N* = 44)	*p*‐value	After PSM CLAR (*N* = 44)	NOSES (*N* = 44)	*p*‐value
Operative time (min)	192 ± 53	188 ± 41	.73	193 ± 52	188 ± 41	.68
Blood loss (mL)	83.6 ± 95.6	48.1 ± 52.7	.009	71.0 ± 55.0	48.1 ± 52.7	.03
Length of abdominal incision (cm)	5.6 ± 0.7	1.2 ± 0.2	<.001	5.7 ± 0.7	1.2 ± 0.2	<.001
Number of dissected lymph nodes	13.1 ± 4.5	12.5 ± 4.2	.38	13.2 ± 4.6	12.5 ± 4.2	.32
Positive lymph nodes (pieces)	0.85 ± 2.1	0.58 ± 1.25	.18	0.78 ± 2.0	0.58 ± 1.25	.56
Positive margin (*N*, %)	0(0)	0(0)	NA	0(0)	0(0)	NA
Intraoperative complications (*N*, %)	0(0)	0(0)	NA	0(0)	0(0)	NA
Histological grade (%)	NA	NA	.94	NA	NA	.92
Well‐differentiated	21(22.8%)	10(22.7%)	NA	10(22.7%)	10(22.7%)	NA
Moderately differentiated	66(71.7%)	30(68.2%)	NA	32(72.7%)	30(68.2%)	NA
Poorly differentiated	5(5.4%)	4(9.1%)	NA	2(4.5%)	4(9.1%)	NA
Histology (*N*, %)	NA	NA	.08	NA	NA	.12
Adenocarcinom	87(94.6%)	41(93.2%)	NA	41(93.2%)	41(93.2%)	NA
Tubular adenocarcinoma	1(1.1%)	1(2.3%)	NA	1(2.3%)	1(2.3%)	NA
Mucinous	4(4.3%)	2(4.5%)	NA	2(4.5%)	2(4.5%)	NA
Use of additional analgesics (*N*, %)	52(56.5%)	16(36.4%)	.03	25(56.8%)	16(36.4%)	.02
VAS score Day 1 postoperatively	3.88 ± 1.15	2.47 ± 0.85	<.001	3.75 ± 1.2	2.47 ± 0.85	<.001
VAS score Day 3 postoperatively	2.91 ± 0.92	1.68 ± 0.63	<.001	2.85 ± 0.95	1.68 ± 0.63	<.001
VAS score Day 5 postoperatively	1.82 ± 0.68	1.28 ± 0.45	<.001	1.80 ± 0.61	1.28 ± 0.45	<.001
Gastrointestinal function recovery time (hour)	52.2 ± 24.7	41.1 ± 15.0	<.001	48.9 ± 16.7	41.1 ± 15.0	.02
Postoperative hospital stay (day)	13.7 ± 5.0	10.8 ± 2.3	<.001	14.2 ± 5.3	10.8 ± 2.3	<.001
Postoperative complications (*N*, %)	18(19.6%)	3(6.8%)	.03	11(25.0%)	3(6.8%)	.01
Anastomotic leakage (*N*, %)	4(4.3%)	2(4.5%)	.97	3(6.8%)	2(4.5%)	.64
Intra‐abdominal infection (*N*, %)	2(2.2%)	0(0)	.51	1(2.3%)	0(0)	.46
Ileus (*N*, %)	1(1.1%)	0(0)	1	1(2.3%)	0(0)	1
Pneumonia (*N*, %)	1(1.1%)	1(2.3%)	1	1(2.3%)	1(2.3%)	1
Pulmonary embolism (*N*, %)	0(0)	0(0)	NA	0(0)	0(0)	NA
Incision‐related complications (*N*, %)	10(10.9%)	0(0)	.02	5(11.4%)	0(0)	.02
Postoperative PFDI‐20	6.92 ± 1.98	6.88 ± 1.75	.60	6.94 ± 2.02	6.88 ± 1.75	.64
Bleeding (*N*,%)	2(2.2%)	0(0)	.51	1(2.3%)	0(0)	.46
Hematoma (*N*, %)	4(4.3%)	0(0)	.12	2(4.5%)	0(0)	.18
Wound infection (*N*, %)	4(4.3%)	0(0)	.12	2(4.5%)	0(0)	.18
Postoperative mortality (*N*, %)	0(0)	0(0)	NA	0(0)	0(0)	NA
Readmission within 30 days (*N*, %)	5(5.4%)	2(4.5%)	.86	2(4.5%)	2(4.5%)	1
1‐year overall survival (%)	94.6	97.7	.42	95.5	97.7	.54
2‐year overall survival (%)	86.9	90.9	.49	88.6	90.9	.68
3‐year overall survival (%)	78.2	84.1	.37	81.8	84.1	.70
4‐year overall survival (%)	73.1	80.8	.30	77.9	80.8	.68
5‐year overall survival (%)	68.8	77.5	.25	74.0	77.5	.72

*Note*: Before PSM: Patient data before propensity score matching. After PSM: Patient data following propensity score matching. Values for ‘Operative time,’ ‘Blood loss,’ ‘Length of abdominal incision,’ ‘Number of dissected lymph nodes,’ ‘Positive lymph nodes,’ and ‘Use of additional analgesics’ are presented as means ± standard deviation (SD). *N*, %: Number and percentage. The *p*‐value was calculated using appropriate statistical tests (independent‐samples T test, Mann–Whitney U test, Chi‐square test, Fisher's exact test, or two‐way repeated measures ANOVA) where appropriate. *p*‐values <.05 are considered statistically significant. “Overall survival” indicates the percentage of patients alive at respective time points post‐surgery (1 year, 2 years, 3 years, 4 years, and 5 years). “Readmission within 30 days” represents the number and percentage of patients readmitted to the hospital within 30 days after discharge. All percentages are calculated based on the total number of patients in the respective groups before and after propensity score matching (PSM).

### 3.3 Postoperative recovery trajectories: Swift revival with NOSES

**FIGURE 2:**
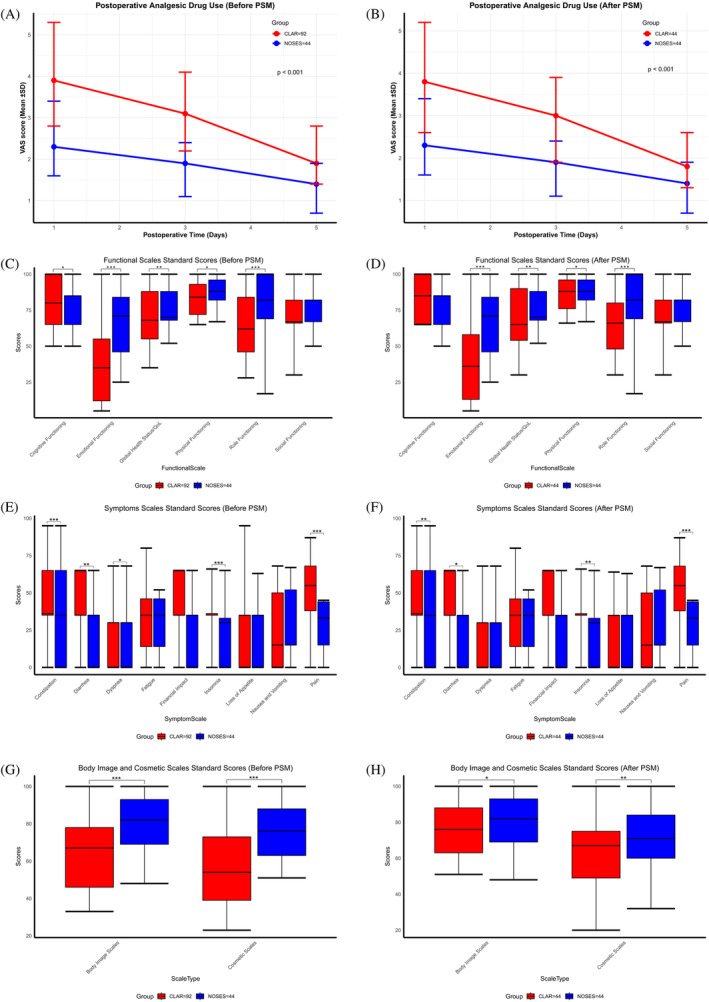
Comparative assessment of short‐term therapeutic outcomes between the two study groups. (A and B) Analgesic medication utilization in the two groups of patients. (A) Postoperative analgesic drug consumption patterns prior to propensity score matching (PSM). (B) Postoperative analgesic drug utilization following PSM. (C–F) Evaluation of quality of life via the EORTC quality of life questionnaire‐core 30 in the two groups of patients. (C) Functional scales–Pre‐PSM. (D) Functional scales–Post‐PSM. (E) Symptom scales–Pre‐PSM. (F) Symptom scales–Post‐PSM. (G and H) Appraisal of body image and cosmetic satisfaction scores. (G) Body image and cosmetic assessment prior to PSM. (H) Body image and cosmetic evaluation following PSM. In all panels, statistical significance levels are denoted as follows: *p < .05, **p < .01, ***p < .001. This figure demonstrates a comprehensive analysis of short‐term curative outcomes within the two study groups. Analgesic drug use, quality of life measures, and body image and cosmetic satisfaction scores are presented, both before and after PSM. Statistical significance indicators highlight noteworthy differences between the groups.

In terms of postoperative outcomes, significant differences were observed between the CLAR and NOSES groups. The use of additional analgesics was significantly higher in the CLAR group compared to the NOSES group (*p* = .02) (Figure 2A,B). Postoperative pain scores, as measured by the VAS, were consistently lower in the NOSES group compared to the CLAR group on days 1, 3, and 5 postoperatively (*p* < .001). The time to gastrointestinal function recovery was significantly shorter in the NOSES group compared to the CLAR group (*p* = .02). Similarly, the postoperative hospital stay was significantly shorter in the NOSES group (*p* < .001). The incidence of postoperative complications was higher in the CLAR group compared to the NOSES group (*p* = .01). Intraoperative endoscopy was uniformly employed in both NOSES and CLAR procedures. The analysis revealed that residual air from endoscopy did not significantly influence the time to return to bowel function, indicating that this factor was effectively mitigated in the comparative evaluation of surgical outcomes. Additionally, evaluation of six‐month postoperative anal function and PFDI‐20 scores indicated no significant differences in anal function between the two groups (Table 3). Furthermore, postoperative symptoms related to the lower urinary tract, lower gastrointestinal tract, and pelvic organ prolapse for the NOSES cohort remained largely unchanged from their preoperative status (Table 4).

**TABLE 3:** Pre‐ and post‐surgery comparison of PFDI‐20 in NOSES patients.

Metric	Pre‐surgery (NOSES, *N* = 44)	Post‐surgery (NOSES, *N* = 44)	*p*‐value
Urinary distress inventory (UDI‐6)	1.65 ± 0.88	1.59 ± 0.92	0.31
Pelvic organ prolapse distress inventory (POPDI‐6)	2.05 ± 0.75	1.88 ± 0.89	0.28
Pelvic floor distress inventory (PFDI‐20)	7.08 ± 1.68	6.65 ± 1.69	0.15
Colorectal‐anal distress inventory (CRADI‐8)	3.48 ± 1.10	3.19 ± 1.18	0.25

*Note*: The PFDI‐20 comprises three components: Pelvic organ prolapse distress inventory 6 (POPDI‐6), colorectal‐anal distress inventory 8 (CRADI‐8), and urinary distress inventory 6 (UDI‐6).

**TABLE 4:** Postoperative Wexner Scores in CLAR and NOSES groups.

	Before PSM			After PSM		
Type of incontinence	CLAR (*N* = 92)	NOSES (*N* = 44)	*p*‐value	CLAR (*N* = 44)	NOSES (*N* = 44)	*p*‐value
Solid	2.31	2.39	.48	2.07	2.39	.13
Liquid	2.06	2.26	.25	2.17	2.26	.44
Gas	3.37	3.29	.62	2.98	3.29	.12
Wears pad	0.06	0.07	.45	0.07	0.07	.75
Lifestyle alteration	2.21	2.06	.14	2.10	2.06	.67

*Note*: Mean values are presented. The *p*‐values indicate statistical differences between the CLAR and NOSES groups before and after propensity score matching (PSM), with values <.05 signifying statistical significance.

When assessing quality of life metrics, the NOSES cohort exhibited significant improvements over the CLR group in functional domains including physical functioning, role functioning, emotional functioning, and global health status. Symptomatically, the NOSES group also reported fewer instances of pain, insomnia, constipation, and diarrhea (Figure 2C–F). Notably, from an esthetic perspective, satisfaction regarding the appearance of the abdominal wall was significantly higher in the NOSES group compared to the CLR group (Figure 2G, H).There were no statistically significant differences in terms of histological grade, histology type, overall survival rates at 1, 2, 3, 4 and 5 years, postoperative mortality, and readmission rates within 30 days between the CLAR and NOSES groups (Table 2).

### 3.4 Postoperative pain: A striking difference

The management of postoperative pain plays a crucial role in patient recovery, comfort, and overall satisfaction. In our comparison of CLAR and NOSES for mid‐rectal cancer treatment, we observed striking differences in postoperative pain experiences. On the first postoperative day, there was a significant difference between the NOSES and CLAR groups. The mean pain scores for the CLAR group were 3.75 ± 1.2, whereas the NOSES group recorded a mean of 2.47 ± 0.85 (*p* < .001).

By the third postoperative day, this disparity persisted. The CLAR group's mean pain scores were 2.85 ± 0.95, contrasting sharply with the NOSES group's scores which were at 1.68 ± 0.63 (*p* < .001). On the fifth postoperative day, the pattern remained consistent. The CLAR group reported a mean score of 1.80 ± 0.61, while the NOSES group's mean stood at 1.28 ± 0.45 (*p* < .001) (Table 2). The data underscores a statistically significant difference in favor of the NOSES group in terms of postoperative pain scores across the evaluated postoperative days, indicating a more comfortable recovery experience with NOSES.

### 3.5 Complication rates: Toward fewer adverse events with NOSES

Postoperative complications represent critical variables in evaluating the effectiveness and safety of surgical procedures. In our comparative study, we noted distinct differences in the complication rates of CLAR and NOSES groups. After PSM, the overall incidence of postoperative complications was significantly lower in the NOSES group (25.0%) compared to the CLAR group (6.8%) (*p* = .006). The NOSES group also demonstrated a lower incidence of specific complications such as anastomotic leakage and incisional infection, though the difference did not reach statistical significance, possibly due to the limited sample size (Table 2).

### 3.6 Long‐term efficacy: Level playing field

**FIGURE 3:**
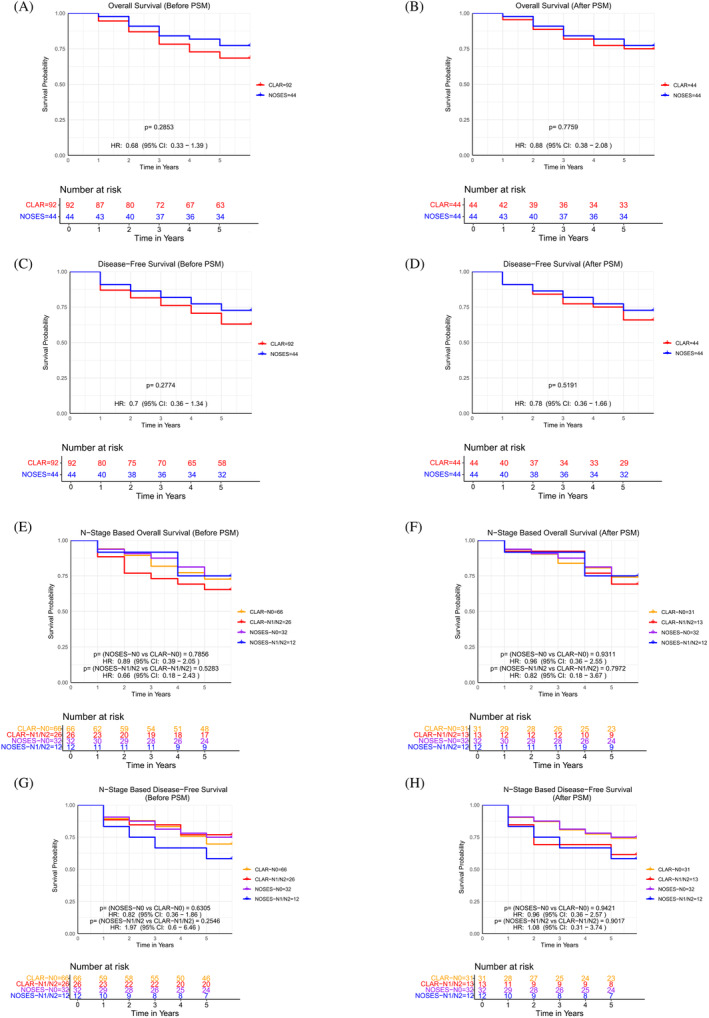
Comparative analysis of long‐term curative outcomes between two groups of patients. (A–D) Overall survival and Disease‐Free survival between two groups. (A) Before PSM overall survival: *p* = .2853, HR: 0.68 (95% CI: 0.33–1.39). (B) After PSM overall survival: *p* = .7759, HR: 0.88 (95% CI: 0.38–2.08). (C) Before PSM disease‐free survival: *p* = .2774, HR: 0.7 (95% CI: 0.36–1.34). (D) After PSM disease‐free survival: *p* = .5191, HR: 0.78 (95% CI: 0.36–1.66). (E–G) Comparison of overall survival and disease‐free survival of patients with different *N* stages in the two groups. (E) Overall survival before PSM: Log‐rank p (NOSES‐N0 vs. CLAR‐N0) = 0.7856, HR: (NOSES‐N0/CLAR‐N0) 0.89 (95% CI: 0.39–2.05); Log‐rank p (NOSES‐N1/N2 vs. CLAR‐N1/N2) = 0.5283, HR: (NOSES‐N1/N2/CLAR‐N1/N2) 0.66 (95% CI: 0.18–2.43). (F) Overall survival after PSM: Log‐rank p (NOSES‐N0 vs. CLAR‐N0) = 0.9311, HR: (NOSES‐N0/CLAR‐N0) 0.96 (95% CI: 0.36–2.55); Log‐rank p (NOSES‐N1/N2 vs. CLAR‐N1/N2) = 0.7972, HR: (NOSES‐N1/N2/CLAR‐N1/N2) 0.82 (95% CI: 0.18–3.67). (G) Disease‐Free survival before PSM: Log‐rank p (NOSES‐N0 vs. CLAR‐N0) = 0.6305, HR: (NOSES‐N0/CLAR‐N0) 0.82 (95% CI: 0.36–1.86); Log‐rank p (NOSES‐N1/N2 vs. CLAR‐N1/N2) = 0.2546, HR: (NOSES‐N1/N2/CLAR‐N1/N2) 1.97 (95% CI: 0.60–6.46). (H) Disease‐Free survival after PSM: Log‐rank p (NOSES‐N0 vs. CLAR‐N0) = 0.9421, HR: (NOSES‐N0/CLAR‐N0) 0.96 (95% CI: 0.36–2.57); Log‐rank p (NOSES‐N1/N2 vs. CLAR‐N1/N2) = 0.9017, HR: (NOSES‐N1/N2/CLAR‐N1/N2) 1.08 (95% CI: 0.31–3.74). This figure demonstrates a comprehensive overview of the long‐term survival outcomes in the two patient groups, both before and after propensity score matching, as well as within different N stages. A *p*‐value of .05 or less is considered statistically significant.

The evaluation of long‐term efficacy is paramount to establish the clinical utility of a surgical procedure. After PSM, both the NOSES and CLAR groups were compared for OS (Overall Survival) and DFS (Disease‐Free Survival), local recurrence rates, and distant metastasis rates. From the longitudinal follow‐up data, we observed no statistically significant differences in both OS and DFS between the two cohorts, as depicted in Figure 3A–D and Table 2. Furthermore, the survival rates, recurrence rates, and distant metastasis rates did not demonstrate any significant disparities between the two groups at the designated follow‐up endpoint, as tabulated in Table 5. A thorough analysis of long‐term follow‐up data, stratified by distinct N stages for both groups, revealed no statistically significant variances in DFS and OS among patients with equivalent N stages (Figure 2E–H). The choice between NOSES and CLAR can be based on factors such as surgical expertise, equipment availability, and patient preference, without compromising the long‐term control of the disease.

**TABLE 5:** Long‐term outcome comparison between CLAR and NOSES groups.

	Before PSM			After PSM		
Outcome	CLAR (*N* = 92)	NOSES (*N* = 44)	*p*‐value	CLAR (*N* = 44)	NOSES (*N* = 44)	*p*‐value
Status at last follow‐up (*N*%)			0.33			0.30
Alive	70(76.1%)	35(79.5%)		30(68.2%)	35(79.5%)	
Dead	22(23.9%)	9(20.5%)		14(31.8%)	9(20.5%)	
Local recurrence (*N*%)	9(9.8%)	3(6.8%)	0.57	4(9.1%)	3(6.8%)	0.74
Distant metastasis (*N*%)	10(10.9%)	5(11.4%)	0.78	5(11.4%)	5(11.4%)	1
Liver metastasis	8(8.7%)	4(9.1%)	/	5(11.4%)	4(9.1%)	/
Lung metastasis	2(2.2%)	2(4.5%)	/	1(2.3%)	2(4.5%)	/

*Note*: Values are presented as counts (*N*) and percentages (%). The *p*‐values represent statistical differences between the CLAR and NOSES groups, before and after propensity score matching (PSM), with values <0.05 indicating statistical significance.

## 4 DISCUSSION

The inception of laparoscopic surgery has significantly redefined the therapeutic landscape of rectal cancer, with a particular focus on augmenting patient recovery and enhancing quality of life.^24^, ^25^ Traditional CLAR, although effective, necessitates an auxiliary abdominal incision, thereby escalating the likelihood of wound infections and hernia complications.^26^, ^27^, ^28^ Emerging as a potential alternative, NOSES promises superior cosmetic outcomes and elevated levels of patient satisfaction.^29^, ^30^, ^31^ This study was designed to perform an in‐depth comparative analysis of these two surgical interventions for mid‐rectal cancer.

The pertinence of comparing these two approaches is amplified by the unique challenges and opportunities posed by mid‐rectal cancers. Unlike their lower rectal counterparts, which are confined by proximity to the anal sphincter,^15^ mid‐rectal cancers permit a wider array of surgical options, making them an ideal cohort for analysis.^12^, ^32^, ^33^

Findings from the current study highlight several advantages of NOSES, particularly concerning postoperative recovery. Patients undergoing NOSES experienced a more rapid return to normal bowel function, reduced length of hospital stay, and lower postoperative pain scores. These results align with existing studies advocating the benefits of NOSES.^3^, ^15^, ^34^ Importantly, preoperative characteristics and operative details appeared comparable between NOSES and CLAR groups, thereby suggesting that the choice of surgical approach could be flexible.

Although no statistically significant disparities in operative times and intraoperative blood loss were observed between the two groups, the postoperative benefits are noteworthy. Specifically, while the time to first flatus was comparable between groups, other recovery metrics such as shorter hospital stay and diminished postoperative pain suggest NOSES might be superior in optimizing patient recovery and comfort. This echoes previous research by.^15^, ^35^, ^36^, ^37^

Oncological safety remains a paramount concern in any surgical intervention for malignancies, demanding rigorous standards and comprehensive outcome assessments.^38^ When considering the overarching goal of oncological safety in surgical interventions for malignancies, it is reassuring to note that the current study found NOSES to be on par with CLAR. A closer scrutiny into possible mechanisms revealed that NOSES's superior recovery metrics could be attributed to reduced surgical trauma and the advantages of intracorporeal anastomosis.^36^, ^39^ Additionally, a minimized wound site could potentially mitigate postoperative pain and infection risk, consistent with the lower complication rates observed in the NOSES group.^40^

Adhering to the paramount principle of oncological safety, no significant differences were identified in lymph node detection or the positive rate of resection margins in postoperative pathology between the two surgical approaches. Moreover, both NOSES and CLAR demonstrated comparable performance in OS and DFS, thereby reinforcing NOSES's alignment with the principle of tumor‐free surgery.

An additional facet of post‐surgical evaluation encompasses the functional outcomes, specifically concerning anal function and overall quality of life in mid‐rectal cancer patients. There have been concerns in the surgical community, as highlighted by Costantino et al. and Nunoo‐Mensah et al.,^41^, ^42^ positing that NOSES might compromise postoperative anal function, primarily due to potential disruption of the anal sphincter's anatomical structure during specimen extraction.^43^ Addressing this concern, our team utilized the Wexner Incontinence Score to gauge postoperative anal sphincter function. The findings were reassuring; no significant difference in postoperative anal function was observed between the two groups. Importantly, NOSES, with its constraints on tumor size and specimen width, seems to provide better regulation and preservation of postoperative anal function.

Evaluating the postoperative quality of life is pivotal, especially in patients undergoing surgical intervention for malignancies. The QLQ‐C30 scale, renowned for its reliability and feasibility, was employed to assess postoperative quality of life in our cohort. Remarkably, the NOSES group outperformed the CLAR group in multiple domains, including global health status, physical function, role function, and emotional function. Moreover, in terms of postoperative symptoms, patients in the NOSES group reported significantly fewer instances of pain, insomnia, constipation, and diarrhea. These outcomes underscore the potential advantages of NOSES, not just from a clinical perspective but also from the holistic well‐being of the patients.

Our data further illuminated a diminished incidence of postoperative complications in the NOSES cohort, corroborating the findings of Zhu et al.^15^ Especially, the diminished rates of anastomotic leakage and incisional infections in the NOSES group emphasize its potential as a safer alternative. This could be tied back to the less invasive nature of NOSES.

The evidence suggests that NOSES is a safe procedure. One of its primary advantages is the absence of an abdominal incision, which understandably leads to heightened patient satisfaction with their abdominal appearance. The omission of an auxiliary incision further ensures that complications often linked to surgical incisions, such as incisional hernias, fat liquefaction, and prolonged healing, are sidestepped. It is worth noting that patients undergoing NOSES reported considerably less postoperative pain, which subsequently reduced their dependency on painkillers. Intriguingly, in light of these numerous benefits, there is no indication of increased hospitalization costs for patients undergoing NOSES.

The observed Length of Stay (LOS) in our study was longer than typical Western standards. This is attributed to the differing healthcare systems and patient management protocols in our region. Extended hospital stays are common here due to socio‐economic factors and patient preferences. This cultural and systemic difference is important to understand the broader implications of LOS in varying healthcare settings.

Hernia assessment was conducted using clinical examinations and imaging at 3 months post‐surgery. We recommend future studies to include extended follow‐up for a more comprehensive understanding of hernia incidence post‐surgery.

Patient BMI was a significant factor in the selection process for NOSES and CLAR procedures. The lower BMI prevalent in our study cohort reflects regional demographic norms and was considered to optimize patient safety and procedural success. This study's findings suggest that while a lower BMI can facilitate certain aspects of laparoscopic surgery, comprehensive patient assessment remains paramount to ensure the procedure's safety and effectiveness. The implications of BMI on surgical outcomes necessitate personalized patient evaluations and highlight the importance of considering demographic characteristics in surgical planning and execution.

Regarding long‐term outcomes, there was alignment between our results and previous findings. There was no discernable difference in parameters such as overall survival and disease‐free survival between NOSES and CLAR. Such outcomes may be more influenced by factors like disease stage and adjuvant therapies than the surgical technique itself.^38^, ^44^

The observed lower anastomotic leak rate in the NOSES group, despite similar anastomotic techniques employed in CLAR, may be attributed to factors such as enhanced visualization and precision, reduced physiological stress, and stringent patient selection. While the precise mechanisms warrant further investigation, these considerations suggest that NOSES may offer intrinsic benefits that contribute to improved anastomotic integrity and patient outcomes. Ongoing and future studies are encouraged to explore these aspects in greater depth to elucidate the definitive factors influencing anastomotic success in NOSES and other minimally invasive procedures.

While NOSES demonstrates substantial promise, it is vital to prioritize patient safety. As highlighted by Stulberg et al.,^45^ the choice of surgical technique should be influenced by individual patient characteristics and surgeon proficiency, rather than technological novelty alone.

From the patient's standpoint, NOSES offers tangible benefits like reduced postoperative pain, improved cosmetic results, and possibly heightened satisfaction.^3^, ^34^, ^46^ As surgical practices gravitate toward minimally invasive approaches, NOSES could eventually become a cornerstone in mid‐rectal cancer care, given the continual advancements and confirmations of its oncological safety.

However, we must integrate NOSES into the broader tapestry of multidisciplinary care for mid‐rectal cancers, ensuring its synergy with other treatments and its long‐term impact on patient care.^47^

Our study contributes to the literature by offering a holistic comparison between NOSES and CLAR in the context of mid‐rectal cancer management. Our results suggest that NOSES, with its postoperative benefits and comparable oncological outcomes, might emerge as a viable alternative to CLAR.

### 4.1 Limitations and future research

This study, albeit rigorous, has limitations inherent to its retrospective design, which may introduce selection biases. Although propensity score matching was deployed to mitigate this limitation, the potential influence of the learning curve on surgical outcomes remains unassessed. Thus, future studies embracing a multicenter, prospective design are warranted to provide a more robust evidence base, offering a comprehensive perspective by leveraging diverse patient populations and varied surgical expertise. Furthermore, it is pertinent to note that our cohort excluded patients who had undergone preoperative chemotherapy or radiation. Recognizing the critical importance of this demographic in colorectal cancer treatment, we advocate for future research to specifically target and assess the efficacy and adaptability of NOSES and CLAR methods following neoadjuvant therapy. This will significantly contribute to the nuanced understanding of these surgical techniques and their role in enhancing patient outcomes in a broader clinical context.

Overall, the implications of this study are far‐reaching, offering a potential paradigm shift in the surgical management of mid‐rectal cancers. As NOSES demonstrates potential advantages in postoperative recovery and complication rates without compromising long‐term oncological outcomes, it could become a preferred option for selected patients.

## 5 CONCLUSIONS

In conclusion, our comparative analysis between CLAR and NOSES for mid‐rectal cancer treatment demonstrated differences in operative details, postoperative recovery trajectories, postoperative pain experiences, complication rates, and long‐term efficacy. NOSES showed advantages in terms of reduced blood loss, shorter incision length, faster recovery, lower postoperative pain, and lower incidence of postoperative complications compared to CLAR. Both procedures exhibited comparable long‐term outcomes, indicating that the choice between the two can be based on individual factors and preferences.

We believe that further prospective studies are needed to validate our findings, alongside exploration of long‐term patient and surgeon perspectives and economic implications. With ongoing advancements and improvements in surgical techniques, alongside increasing patient expectations, it is inevitable that more innovative procedures such as NOSES will continue to be developed and scrutinized. It is our responsibility as clinicians and researchers to rigorously assess these new techniques, ensuring they meet or exceed current standards in terms of both patient satisfaction and treatment outcomes. This study forms part of that ongoing dialog, and we hope that it will provide a basis for further exploration and discussion in the field. These investigations will provide a holistic understanding of the true potential of NOSES in the management of mid‐rectal cancers, and whether it can be widely adopted as a safe and effective treatment method.

## AUTHOR CONTRIBUTIONS

Shan Muhammad, Zheng Jiang, and Tao Fan contributed to formal analysis, writing‐original draft, data curation, and editing. Shan Muhammad, QingChao Tang, Yang Hai, Sundas Bint E Ehsan, Maimoona Bilal, and Albina A. Zubayraeva were involved in project administration, resource management, writing‐original draft, data curation, methodology, and supervision. Shan Muhammad, Zheng Jiang, and Albina A. Zubayraeva collaborated on data curation, methodology, resource management, software, and supervision. Shan Muhammad, Sundas Bint E Ehsan, and Maimoona Bilal contributed to the methodology. Lastly, Shan Muhammad, YiBo Gao, and Jie He undertook conceptualization, supervision, and writing review & editing, while YiBo Gao and Jie He handled visualization and funding acquisition.

## FUNDING INFORMATION

This study was supported by National Key R&D Program of China (2020YFC2006400, 2020AAA0109500), the National Natural Science Foundation of China (82122053 to YiBo Gao, 82188102 to Jie He), CAMS Initiative for Innovative Medicine (2021‐I2M‐1‐067 to YiBo Gao), Non‐profit Central Research Institute Fund of Chinese Academy of Medical Sciences (2021‐RC310‐020 to YiBo Gao) and Key‐Area Research and Development Program of Guangdong Province (2021B0101420005 to YiBo Gao).

## CONFLICT OF INTEREST STATEMENT

The authors declare no conflict of interest.

## ETHICS STATEMENT

The study was conducted in accordance with the Declaration of Helsinki, and approved by the Ethics Committee of the National Cancer Center/National Clinical Research Center for Cancer/Cancer Hospital, affiliated with the Chinese Academy of Medical Sciences & Peking Union Medical College in Beijing, China (CAMS). In this study, due to its analytical nature, a separate ethics review was not required.

## Supporting information


**Supplementary Material 1**: These supplementary videos offer a comprehensive visual guide to the NOSES surgical procedure, highlighting each significant step with precision and clarity.

Supplementary Video 1: Mobilization of the descending and sigmoid colon.

This video illustrates the crucial first step in the NOSES surgical procedure, demonstrating the meticulous mobilization of the descending and sigmoid colon.

Supplementary Video 2: Clipping of the inferior mesenteric artery using hem‐o‐lock clips.

In this video, the surgical team showcases the precise technique of clipping the inferior mesenteric artery with Hem‐o‐Lock clips, a key maneuver in the NOSES approach.

Supplementary Video 3: Dissection of the mesorectum following total mesorectal excision (TME).

This video provides an in‐depth view of the mesorectal dissection, a critical component of the NOSES procedure, following the principles of Total Mesorectal Excision (TME).

Supplementary Video 4: Delivery of the anvil in the sigmoid and resection of the rectum using the linear stapler echelon 60.

Watch as the surgical team delivers the anvil into the sigmoid and performs rectal resection using the advanced Linear Stapler Echelon 60.

Supplementary Video 5: Removal of the anvil connection rod at the distal end of the sigmoid colon.

This video showcases the precise removal of the anvil connection rod at the distal end of the sigmoid colon, ensuring a seamless transition in the surgical process.

Supplementary Video 6: Transanal extraction of the opened distal rectal stump for smooth specimen extraction.

Observe the meticulous transanal extraction of the opened distal rectal stump in this video, a step that facilitates smooth specimen extraction.

Supplementary Video 7: Extracorporeal resection of the rectal specimen and inspection for adequate distal margin.

This video captures the extracorporeal resection of the rectal specimen, followed by a thorough inspection to ensure an adequate distal margin.

Supplementary Video 8: Passage of the head of the circular stapler through the anal orifice and end‐to‐end double‐stapled anastomosis.

Witness the precise passage of the circular stapler through the anal orifice, culminating in an end‐to‐end double‐stapled anastomosis.

**Supplementary Video 9: Positioning of the post‐operative peritoneal drainage tube near the anastomosis site**.

In this video, the surgical team demonstrates the placement of the post‐operative peritoneal drainage tube near the site of the anastomosis, a crucial step in the NOSES procedure.

**Data S2:** Supporting Information.

**Supplementary Material 3:** Wexner incontinence score.

## Data Availability

All data generated during this study are comprehensively presented within the manuscript in the form of figures, tables, and multimedia. Any additional data related to this research are available as supplementary material accompanying the manuscript.
