# Supplementary material for: Advancing mid‐rectal cancer surgery: Unveiling the potential of natural orifice specimen extraction surgery in comparison to conventional laparoscopic‐assisted resection
Source: Cancer Rep (Hoboken). 2024 May 4;7(5):e2003. doi: 10.1002/cnr2.2003 (PMC11069103; doi:10.1002/cnr2.2003)
Supplement: Supplementary file 2 — Data S2: Supporting Information. [file CNR2-7-e2003-s002.docx]

**The Body Image Questionnaire**

***Please rate the following statements according to your experiences since the operation:***

**1.To what extent has your body image been affected by the surgery?**

1 = Not at all

2 = Slightly

3 = Moderately

4 = Significantly

**2.Do you believe the surgery has caused damage to your body?**

1 = Not at all

2 = Slightly

3 = Moderately

4 = Significantly

**3.Have you noticed a decrease in your perceived attractiveness due to your illness or treatment?**

1 = Not at all

2 = Slightly

3 = Moderately

4 = Significantly

**4.Do you feel that your sense of femininity/masculinity has been reduced as a consequence of your illness or treatment?**

1 = Not at all

2 = Slightly

3 = Moderately

4 = Significantly

**5.Has it become challenging to view yourself unclothed?**

1 = Not at all

2 = Slightly

3 = Moderately

4 = Significantly

**6.Please rate your satisfaction with your incisional scar on a scale from 1 to 7:**

1 = Very unsatisfied

2 = Unsatisfied

3 = Somewhat unsatisfied

4 = Neutral

5 = Somewhat satisfied

6 = Satisfied

7 = Very satisfied

**7.How would you describe the appearance of your incisional scar on a scale from 1 to 7?**

1 = Revolting

2 = Unattractive

3 = Somewhat unattractive

4 = Neutral

5 = Somewhat attractive

6 = Attractive

7 = Beautiful

**8.On a scale from 1 to 10, with 1 being the worst and 10 being the best, please rate your incisional scar.**

**9.Have you experienced any discomfort or pain related to your incisional scar?**

1 = None

2 = Mild

3 = Moderate

4 = Severe

**10.How have your body image and feelings about your scar evolved over time since the surgery?**

1 = Significantly worsened

2 = Somewhat worsened

3 = Remained stable

4 = Improved somewhat

5 = Improved significantly

***Thank you for participating in this questionnaire. Your responses are valuable for our research.***
