# Supplementary material for: Advancing mid‐rectal cancer surgery: Unveiling the potential of natural orifice specimen extraction surgery in comparison to conventional laparoscopic‐assisted resection
Source: Cancer Rep (Hoboken). 2024 May 4;7(5):e2003. doi: 10.1002/cnr2.2003 (PMC11069103; doi:10.1002/cnr2.2003)
Supplement: Supplementary file 3 — Supplementary Material 3: Wexner incontinence score. [file CNR2-7-e2003-s003.docx]

**Supplementary Material 3: Wexner Incontinence Score**

| **Type of Incontinence** | **Frequency** | | | | |
| --- | --- | --- | --- | --- | --- |
|  | **NEVER** | **Rarely** | **Some-**  **times** | **Usually** | **Always** |
| **Solid** | 0 | 1 | 2 | 3 | 4 |
| **Liquid** | 0 | 1 | 2 | 3 | 4 |
| **Gas** | 0 | 1 | 2 | 3 | 4 |
| **Wears pad** | 0 | 1 | 2 | 3 | 4 |
| **Lifestyle alteration** | 0 | 1 | 2 | 3 | 4 |

**Scoring:**

0 = Perfect

20 = Complete Incontinence

**Frequency:**

Never = 0 (never).

Rarely ≤ l/month.

Sometimes ≤ l/week, ≥ l/month.

Usually ≤ l/day, ≥ l/week.

Always ≥ l/day.

***Note:*** This table presents the Wexner Incontinence Score, which assesses different types of incontinence based on frequency, ranging from never to always. The scores provide insights into the level of incontinence experienced by individuals, with 0 indicating no incontinence and 20 signifying complete incontinence. The frequency categories further classify the severity of incontinence based on different time intervals.
